# Supplementary material for: Quality Characteristics of Vegan Mayonnaise Produced Using Supercritical Carbon Dioxide-Processed Defatted Soybean Flour
Source: Foods. 2024 Apr 11;13(8):1170. doi: 10.3390/foods13081170 (PMC11048837; doi:10.3390/foods13081170)
Supplement: Supplementary file 1 [file foods-13-01170-s001.zip › foods-2904516-supplementary.pdf]

Table S1. Proximate composition of control soy flour (CSF), defatted soy flour treated with SC-CO_2 (DSF), and egg yolk (EY).

Table S2. pH of mayonnaises prepared with control soy flour (CSF), defatted soy flour treated with SC-CO_2 (DSF), and egg yolk (EY).

Table S3. Particle size diameter (μm) of mayonnaises prepared with control soy flour (CSF), defatted soy flour treated with SC-CO_2 (DSF), and egg yolk (EY).

Table S4. Particle size analysis of mayonnaise with different emulsifiers at different concentrations.

Table S1

Proximate composition (%)	CSF	DSF	EY
Moisture	7.41±0.65^{c}	3.30±0.08^{a}	3.48±0.24^{b}
Ash	5.41±0.29^{b}	6.26±0.14^{c}	3.94±0.21^{a}
Protein	38.66±0.66^{b}	48.93±0.82^{c}	36.20±0.74^{a}
Fat	18.95±0.95^{b}	0.51±0.37^{a}	54.27±0.56^{c}
Carbohydrate	29.50±1.03^{b}	41.00±0.15^{a}	2.11±0.64^{c}

CSF: control soy flour; DSF: defatted soybean flour treated with SC-CO_2; EY: egg yolk.

All values are mean ± SD (n=3); Means with different superscripts letters within the same column are significantly different ($p < 0.05$) using Duncan's test.

Table S2

pH	% Addition of Emulsifier				
	0.5	1	1.5	2	3
**CSF**	3.83±0.01$^{bA5)}$	3.99±0.02^{bB}	4.11±0.01^{bC}	4.20±0.01^{bD}	4.34±0.00^{bE}
**DSF**	3.61±0.02^{aA}	3.80±0.01^{aB}	3.92±0.00^{aC}	4.03±0.01^{aD}	4.16±0.02^{aE}
**EY**	4.17±0.01^{cA}	4.31±0.01^{cB}	4.42±0.00^{cC}	4.58±0.01^{cD}	4.67±0.01^{cE}

CSF: control soy flour; DSF: defatted soybean flour treated with SC-CO_2; EY: egg yolk.

All values are mean ± SD (n=3). Different letters (a–e for the samples at different emulsifiers within the same column, A–E for the samples at different additive levels within the same row) indicate significant differences ($p < 0.05$) by Duncan's test.

Table S3

Particle size diameter (μm)	% addition of emulsifier				
	**0.5**	**1**	**1.5**	**2**	**3**
**CSF**	53.35±0.12^{cE}	45.85±0.25^{cD}	42.46±0.30^{cC}	39.38±0.32^{cB}	33.01±0.22^{cA}
**DSF**	35.84±0.1^{bE}	32.90±0.04^{bD}	28.64±0.05^{bC}	24.24±0.20^{bB}	12.02±0.26^{bA}
**EY**	28.98±0.03^{aE}	27.70±0.12^{aD}	23.33±0.21^{aC}	15.96±0.08^{aB}	8.3±0.16^{aA}

CSF: control soy flour; DSF: defatted soy flour treated with SC-CO_2; EY: egg yolk.

Different letters (a-e for the samples at different emulsifiers within the same column, A-E for the samples at different additive levels within the same row) indicate significant differences (p < 0.05) by Duncan's test.

Table S4. Particle size analysis of mayonnaise with different emulsifiers at different concentrations.

$d_{4,3}$	Different addition of emulsifier (%)				
	**0.5**	**1**	**1.5**	**2**	**3**
**CSF**	53.35±0.12$^{cE1)}$	45.85±0.25^{cD}	42.46±0.30^{cC}	39.38±0.32^{cB}	33.01±0.22^{cA}
**DSF-SC**	35.84±0.1^{bE}	32.90±0.04^{bD}	28.64±0.05^{bC}	24.24±0.20^{bB}	12.02±0.26^{bA}
**EY**	28.98±0.03^{aE}	27.70±0.12^{aD}	23.33±0.21^{aC}	15.96±0.08^{aB}	8.3±0.16^{aA}

[1)]Lowercase letters (a-c) indicate significant differences between different emulsifiers within the same column, while uppercase letters (A-E) indicate significant differences between different additive levels within the same row. Different letters denote significant differences among samples ($p < 0.05$)
